# Beyond Aqueductal Stenosis: A Case of an Atypical Teratoid/Rhabdoid Tumor Presenting With Neonatal Hydrocephalus

**DOI:** 10.7759/cureus.100271

**Published:** 2025-12-28

**Authors:** Jochen Gerstner Saucedo, Yudel Tamayo, Tyler Herrington, Sakir H Gultekin, Heather McCrea, Sameer Samtani, Gaurav Saigal

**Affiliations:** 1 Diagnostic Radiology, University of Colorado Anschutz Medical Campus, Aurora, USA; 2 Diagnostic Radiology, University of Miami Miller School of Medicine, Jackson Memorial Hospital, Miami, USA; 3 Pathology, University of Miami Miller School of Medicine, Miami, USA; 4 Pediatric Neurosurgery, University of Miami Miller School of Medicine, Jackson Memorial Hospital, Miami, USA; 5 Radiology, University of Miami Health System/Jackson Memorial Hospital, Miami, USA; 6 Radiology, University of Miami, Miami, USA

**Keywords:** atypical teratoid/rhabdoid tumor (atrt), embryonal brain tumor, endoscopic third ventriculostomy (etv), neonatal hydrocephalus, rhabdoid tumor predisposition syndrome (rtps1), smarcb1 mutation

## Abstract

An atypical teratoid/rhabdoid tumor (ATRT) is a rare and highly aggressive embryonal tumor in the central nervous system, with the highest incidence in infants. Neonatal cases frequently experience delayed diagnosis due to nonspecific symptoms, with imaging findings that may mimic more prevalent congenital anomalies such as aqueductal stenosis. We report a case of a full-term male neonate with prenatal findings of severe lateral and third ventriculomegaly, initially misdiagnosed to be due to an obstructive etiology due to hemorrhage in the fourth ventricle/aqueduct. Later, on a postnatal MRI, a non-enhancing hemorrhagic mass was suspected to be arising from the tectum, which on histopathology was confirmed to be an ATRT with loss of SMARCB1 (INI1) expression.

## Introduction

An atypical teratoid/rhabdoid tumor (ATRT) is a rare and aggressive embryonal tumor of the central nervous system (CNS) that mainly affects infants (<one year) and young children (1-3 years) [1-3]. It is caused by a loss of function of the SMARCB1 (also known as INI1) gene, a core SWI/SNF tumor suppressor, either as a somatic mutation or as part of the rhabdoid tumor predisposition syndrome type 1 (RTPS1). The annual incidence in the United States is approximately 0.07-0.09 per 100,000 children under 15 years, highest in children under one year of age (up to 0.58 per 100,000) [2,4-6].

Clinical signs and symptoms of ATRTs are diverse and include signs of increased intracranial pressure (vomiting, lethargy, macrocephaly, irritability, drowsiness), which are the most frequent clinical presentations of ATRTs in infants and young children under three years, while focal neurological deficits, cranial nerve palsies, ataxia, and seizures are less common but may occur depending on the tumor location [7]. Infratentorial tumors more commonly cause ataxia and hydrocephalus, while supratentorial tumors may lead to seizures or focal deficits. Imaging features are nonspecific but typically reveal heterogeneous, iso- or hypointense masses on T1- and T2-weighted MRI with or without enhancement, necrosis, or hemorrhage [8]. Variable features and a broad differential diagnosis complicate early detection and lead to frequent misidentification [5,9]. 

There are no specific prenatal ultrasound or biophysical profile findings that reliably predict ATRTs. Most ATRTs present postnatally with nonspecific signs of raised intracranial pressure or mass effect, and the tumors are typically not detected on routine obstetric imaging.

The only robust pre-symptomatic predictor identified to date is alteration of the SMARCB1 (INI1) gene [2]. Germline SMARCB1 mutations cause rhabdoid tumor predisposition syndrome and markedly increase the risk of ATRTs and other malignant rhabdoid tumors in early childhood, so genetic testing is recommended in families with a history of rhabdoid tumors or synchronous/extracranial rhabdoid tumors [2]. In these high-risk infants, postnatal surveillance MRI has been proposed rather than prenatal ultrasound-based screening, because CNS ATRTs develop rapidly and may not be visible prenatally.

Once ATRTs are diagnosed, DNA methylation profiling (ATRT-TYR, ATRT-SHH, ATRT-MYC) and clinical factors (age <one year, metastatic disease, synchronous tumors) help with prognostic stratification, but they do not provide a prenatal imaging marker [10-12].

We report a case of a neonatal ATRT presenting with severe lateral and third ventriculomegaly, initially misdiagnosed as obstructive hydrocephalus due to hemorrhage.

## Case presentation

We present the case of a male neonate born at 37 weeks and 1 day via spontaneous vaginal delivery to a 34-year-old G4P3 mother. Pregnancy was notable for gestational diabetes and chronic hypertension, with induction of labor due to the latter. Prior to delivery, a routine prenatal ultrasound performed at 32 weeks identified severe bilateral ventriculomegaly with a dangling choroid plexus.

**Figure 1:**
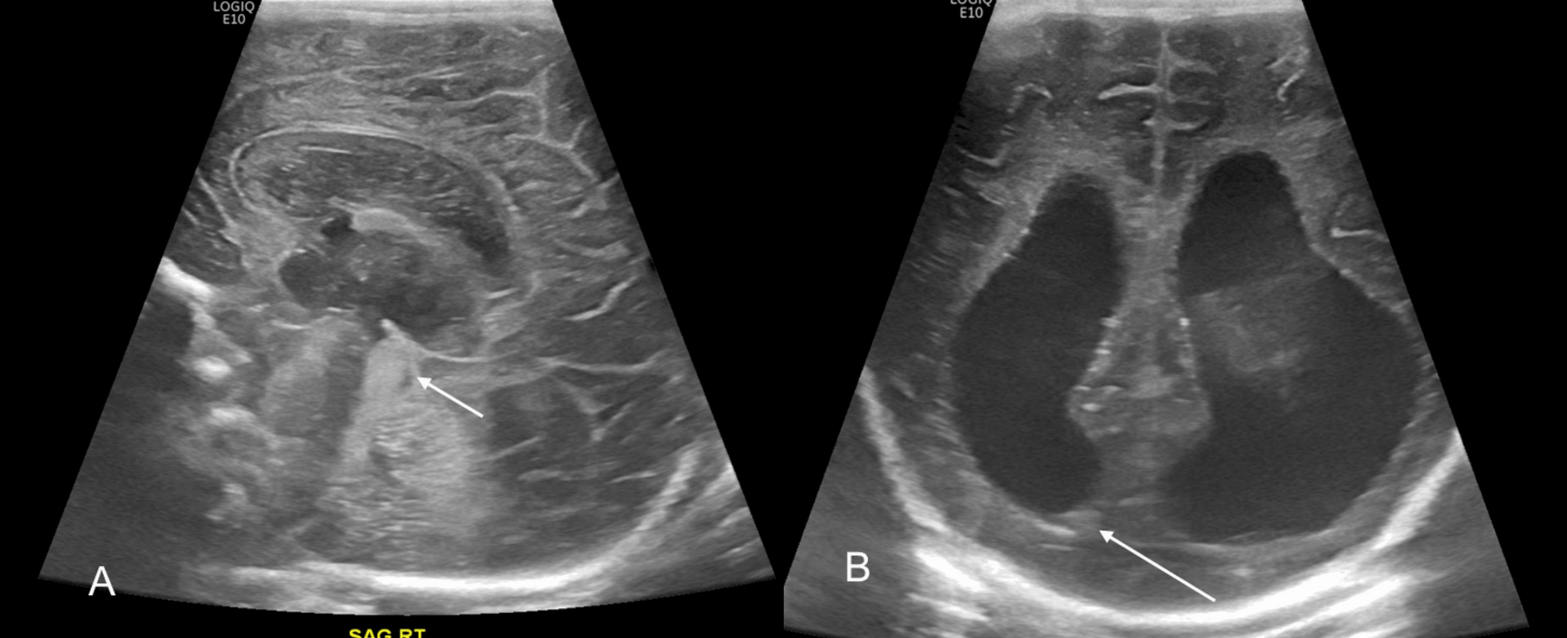
Midline sagittal (A) and coronal (B) images from echoencephalogram performed at day 1 of life, demonstrating a focus of increased echogenicity in the aqueduct/fourth ventricle (short white arrow) and a minimal amount of layering intraventricular hemorrhage in the right ventricle (long white arrow). Moderate to severe hydrocephalus of the lateral and third ventricles is noted.

**Figure 2:**
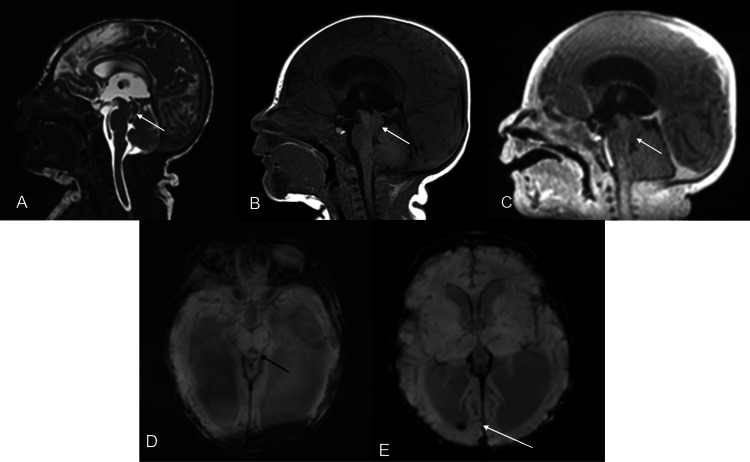
Sagittal T2 CISS (A), sagittal T1W pre (B) and postcontrast (C) MR images of the brain demonstrating a nonenhancing isointense T1/T2 lesion noted in the aqueduct, extending into the upper fourth ventricle (short white arrows). No restricted diffusion was noted (not shown). There is susceptibility noted within the lesion (black arrow) as well as a small amount of right intraventricular hemorrhage (long white arrow) on the SWI images (D, E) within the dilated ventricles. CISS: Constructive interference in steady state; MR: magnetic resonance

Postnatally, the patient remained neurologically stable but developed progressive macrocephaly. On day 1 of life, an echoencephalogram demonstrated ventriculomegaly with a focus of increased echogenicity at the aqueduct extending to the fourth ventricle, suspicious for hemorrhage with resultant obstructive hydrocephalus. A brain MRI on day 3 of life revealed a lobulated soft tissue density within the sylvian aqueduct felt, most likely to reflect blood products because of susceptibility artifact in the aqueduct, fourth ventricle, and occipital horn of the right lateral ventricle. A follow-up FAST MRI on day 16 demonstrated further worsening hydrocephalus without any expected evolution of the hemorrhage. A repeat MRI of the brain with contrast was performed on day 18, which demonstrated what appeared to be a non-enhancing tectal mass extending to the fourth ventricle (Figures 1, 2). The patient was then scheduled for endoscopic third ventriculostomy with choroid plexus cauterization (ETV/CPC) as the initial intervention to treat hydrocephalus.

**Figure 3:**
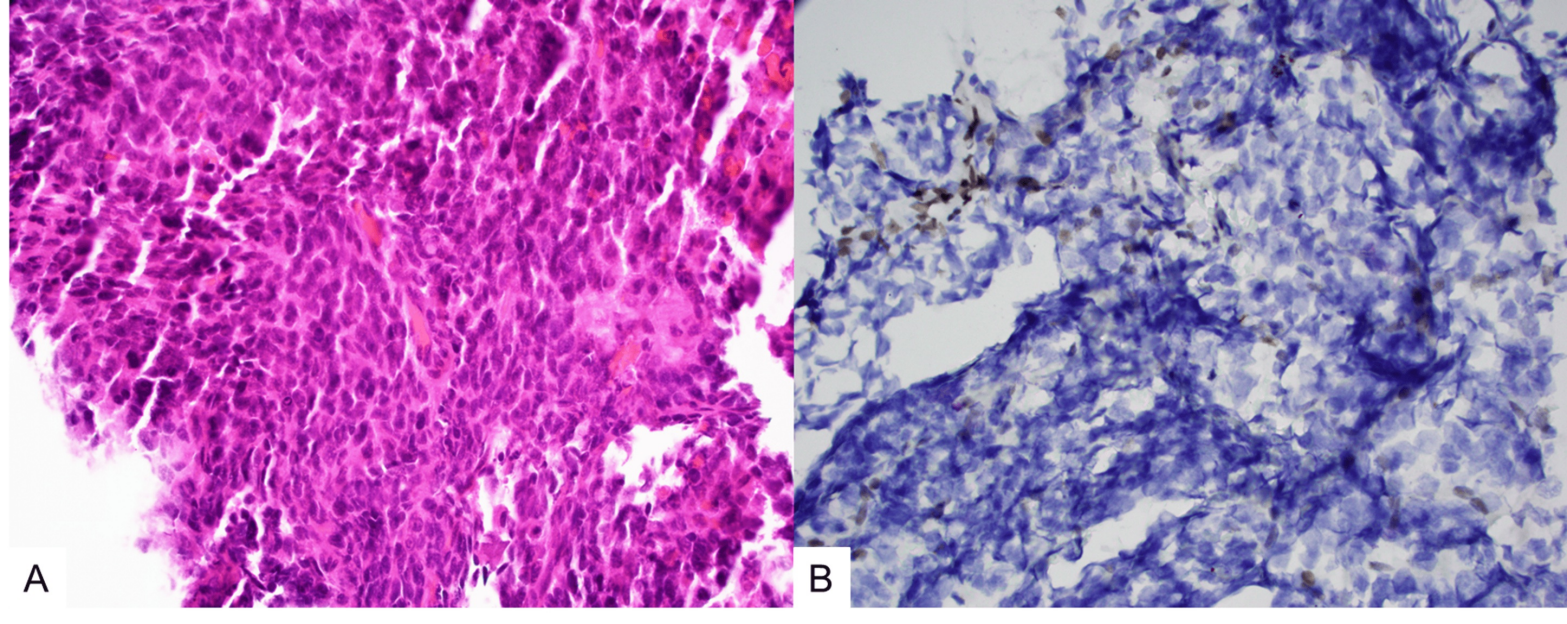
Histopathologic findings. (A) Hematoxylin and eosin stain (H&E, ×400) demonstrates a highly cellular neoplastic tissue with poorly differentiated cells with large nuclei and coarse chromatin. (B) Immunohistochemical stain for INI-1 (BAF47/SMARCB1, ×400) shows complete loss of SMARCB1 protein in tumor cells with retained expression in blood vessel endothelial cells only (upper left).

During the procedure (day 19), a small lesion was unexpectedly encountered extending from the wall of the third ventricle, blocking the intended site for ventriculostomy at the floor. This lesion was not visible on preoperative MRI. Its presence precluded safe completion of the endoscopic third ventriculostomy, but given its appearance concerning for leptomeningeal spread of the tumor, endoscopic biopsy was performed. The patient had a ventricular shunt placed on day 26 to treat hydrocephalus. Histopathologic evaluation revealed a malignant embryonal tumor with features concerning for either pineoblastoma or an ATRT (Figure 3). Immunohistochemical staining demonstrated loss of nuclear INI1 (SMARCB1) expression, confirming the diagnosis of an ATRT. Whole genome sequencing was performed, and genetic testing revealed a heterozygous germline SMARCB1 mutation, consistent with RTPS1.

A staging workup by pediatric hematology/oncology included MRI of the cervical, thoracic, and lumbar spine with contrast, MIBG imaging, and urine HVA/VMA to exclude metastatic or neuroblastoma-related disease. Serum AFP was 5,842 early in the evaluation; CSF cytology later showed only rare atypical cells in a bloody background.

Given the neonatal age and the concern for metastatic (M1) disease, the patient was started on induction chemotherapy per institutional protocol 02-294, which corresponds to the intensive multimodality ATRT regimen [13]. In our center, this included multi-agent systemic chemotherapy with vincristine, cisplatin, cyclophosphamide, and etoposide, plus intrathecal chemotherapy, with doses reduced for age, and radiotherapy omitted because of the patient’s age and M1 status.

**Figure 4:**
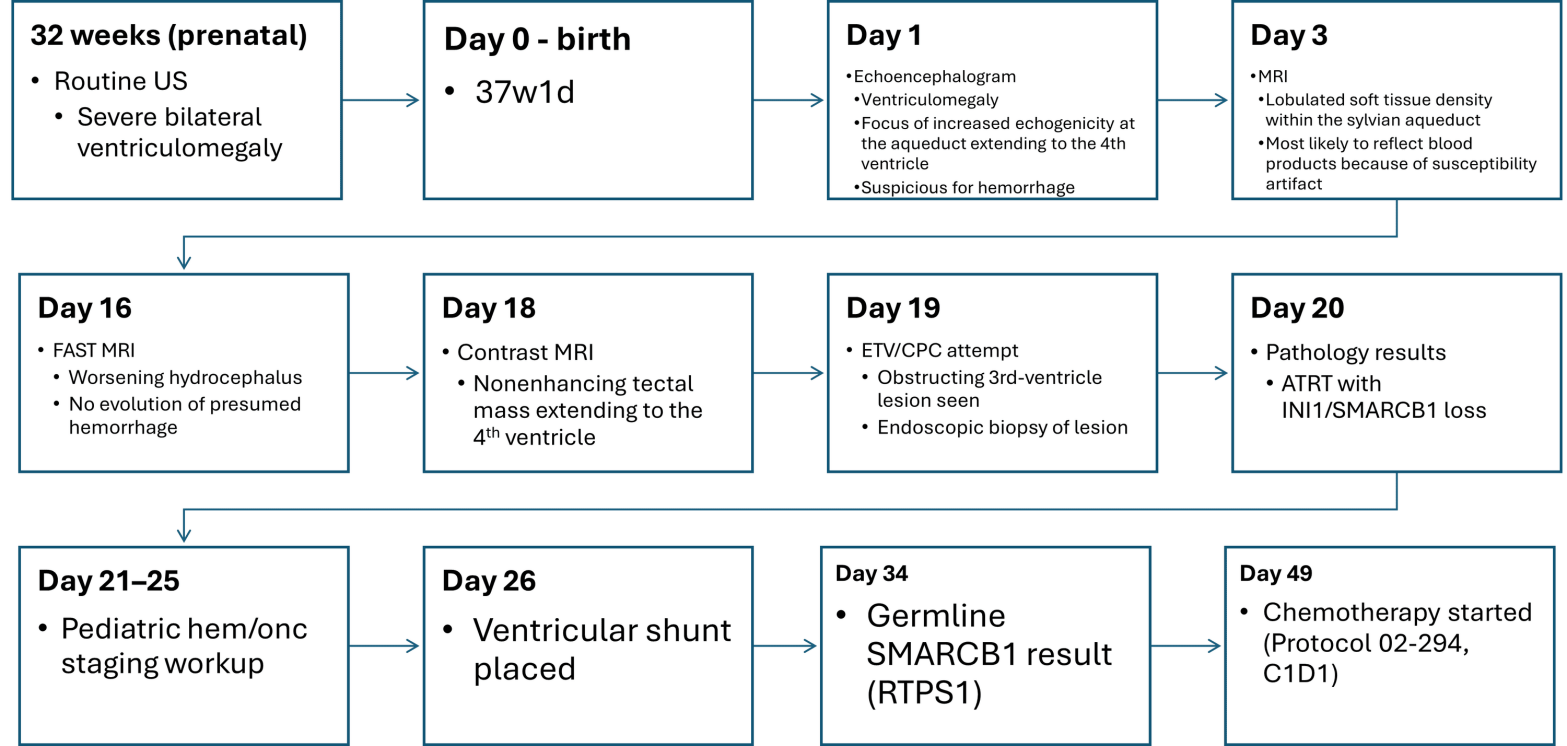
Timeline of the clinical course—from prenatal detection of ventriculomegaly through postnatal imaging, ETV/CPC attempt and biopsy, pathologic confirmation of ATRT with INI1/SMARCB1 loss, genetics (RTPS1), CSF diversion, and initiation/escalation of chemotherapy (02-294 → ACNS0333). ETV: Endoscopic third ventriculostomy; CPC: choroid plexus cauterization

After approximately six weeks of induction chemotherapy per protocol 02-294 (with intrathecal therapy), interval MRI showed only partial improvement with residual disease, which was judged an inadequate response for this high-risk neonatal ATRT. Because of this, the oncology team discontinued 02-294 and escalated therapy to the COG ATRT regimen ACNS0333. As part of ACNS0333, the patient proceeded to high-dose chemotherapy followed by autologous stem-cell rescue, with the first transplant performed after the first high-dose block (approximately five weeks after escalation). The patient is currently receiving this regimen. At the latest follow-up, the patient remained neurologically stable, with no new lesions identified on serial surveillance imaging. On the WHO head-circumference standards for boys, the median at ~6 months is ~43 cm; this patient’s 40 cm (Z −3.15; 0.08th percentile) indicates a markedly low occipito-frontal circumference. Ongoing growth monitoring and neurodevelopmental follow-up are being managed by the primary care team. Figure 4 summarizes the case chronology from prenatal ultrasound through CSF diversion, diagnostic confirmation, and initiation of systemic therapy.

## Discussion

An ATRT is a rare and highly aggressive tumor of the CNS that primarily affects children. It mostly occurs in children under three years old, making up about 1-2% of all pediatric brain tumors [9]. The incidence is especially high among infants and young children, with the highest rates seen in those under two years old. ATRT is classified as a CNS embryonal tumor with a highly malignant course and poor prognosis, marked by rapid progression and an increased risk for dissemination through cerebrospinal fluid. Because imaging characteristics can be ambiguous, especially in newborns, diagnosis often relies on histology and immunohistochemistry [3].

This is an unusual case of a neonate presenting with hydrocephalus in whom the initial prenatal and early postnatal imaging suggested obstructive ventriculomegaly due to echogenic material within the aqueduct, most consistent with hemorrhage causing functional aqueductal obstruction. Subsequent MRI demonstrated a lobulated, nonenhancing soft-tissue lesion at the level of the tectum/aqueduct with associated susceptibility foci, which explained the persistent obstruction and lack of expected hemorrhage evolution. At the time of endoscopic third ventriculostomy, a mass was identified at the floor of the third ventricle and biopsied, confirming ATRT. The aqueductal focus seen on early studies was therefore understood to represent the same obstructing process rather than isolated congenital aqueductal stenosis or simple hemorrhage.

In neonates with obstructive hydrocephalus, the differential diagnosis of a tectal lesion includes tectal gliomas, hemorrhage, and less common embryonal tumors like ATRT. Tectal gliomas are typically well-defined, hyperintense on T2-weighted images, and do not show enhancement, often displaying minimal mass effect or diffusion restriction [14]. In contrast, ATRTs usually present as heterogeneous, enhancing masses that may contain necrosis, hemorrhage, or calcification, and often show restricted diffusion [15]. This case was unusual because the lesion did not enhance and initially resembled a benign appearing hemorrhage, which delayed the diagnosis. 

Magnetic resonance spectroscopy can offer further diagnostic insights: ATRTs usually reveal elevated choline levels, notable peaks of lipid and lactate, and significantly reduced N-acetylaspartate, indicating a high-grade tumor. In contrast, tectal gliomas often lack these aggressive metabolic features [16]. To distinguish an ATRT from an evolving hemorrhage, particularly in neonates, serial imaging can be useful. A hemorrhage would be expected to evolve/reduce with time. Lack of evolution, particularly with an increase in size, would be concerning for a more aggressive pathology, as in this case. In addition, characteristics such as peritumoral edema, peripheral cysts, and infiltrative margins would suggest a neoplastic etiology and should lead to considering ATRTs in the differential diagnosis [15].

To our knowledge, there are no previously published case reports or series describing a neonatal ATRT presenting as a nonenhancing tectal or aqueductal lesion, initially misdiagnosed as aqueductal stenosis or intraventricular hemorrhage, with diagnosis only made intraoperatively or on biopsy after failed ventriculostomy. While diagnostic confusion with other pathologies, such as PNET or NF-associated tumors, has been described, no report has detailed a presentation as subtle as in this case [17-19]. This case may therefore represent the first reported instance of an ATRT masquerading as a non-evolving hemorrhage with an initially non-visible lesion on MRI, highlighting the need to consider neoplasia even in the absence of classic imaging findings.

## Conclusions

In neonates with progressive ventriculomegaly, a lobulated, nonenhancing tectal or aqueductal lesion that shows susceptibility foci in the aqueduct and fourth ventricle, but does not show the typical temporal evolution of hemorrhage, should prompt consideration of ATRT. In this case, persistent aqueductal obstruction, tectal displacement, worsening hydrocephalus on FAST MRI, and subsequent identification of a nonenhancing tectal mass were the key radiologic clues. The initially non-enhancing, subtle lesion limits this single-case observation; nonetheless, nonresolving obstructive ‘hemorrhage’ in this location warrants repeat MRI and early biopsy consideration to avoid treatment delays. ATRTs in this age group can mimic obstructive congenital or hemorrhagic etiologies, and early recognition is important to obtain tissue diagnosis, initiate appropriate oncologic therapy, and pursue SMARCB1-related genetic counseling.
